# Dicentrine, an Aporphine Alkaloid From *Ocotea langsdorffii*, Induces Mitochondrial Dysfunction and Oxidative Stress in *Leishmania (L.) infantum*

**DOI:** 10.1002/cbdv.71575

**Published:** 2026-08-09

**Authors:** Camila S. de Freitas, Eduardo Oliveira S. da Silva, Daniela C. Tristão, Guilherme M. Antar, Daniela P. Lage, Dóris M. Abrão, Luciana M. Ribeiro Antinarelli, Elaine S. Coimbra, João Henrique G. Lago, Eduardo A. F. Coelho

**Affiliations:** ^1^ Post‐graduation Program in Health Sciences ‐ Infectiology and Tropical Medicine, Medicine Faculty Federal University of Minas Gerais Belo Horizonte Minas Gerais Brazil; ^2^ Center of Natural Sciences and Humanities Federal University of the ABC Santo Andre Sao Paulo Brazil; ^3^ Department of Agricultural and Biological Sciences Federal University of Espirito Santo São Matheus Espirito Santo Brazil; ^4^ Department of Parasitology, Microbiology and Immunology, Institute of Biological Sciences Federal University of Juiz de Fora Juiz de Fora Minas Gerais Brazil

**Keywords:** antileishmanial action, dicentrine, mitochondrial membrane, *Ocotea langsdorffii*, plasma membrane

## Abstract

Dicentrine, an aporphine alkaloid isolated from *Ocotea langsdorffii*, was evaluated for its in vitro antileishmanial activity against *Leishmania (L.) infantum*, *Leishmania (L.) amazonensis*, and *Leishmania (V.) braziliensis*. Cytotoxicity was assessed in murine peritoneal macrophages, and the mechanism of action was investigated in *L. (L.) infantum* promastigotes by evaluating mitochondrial membrane potential (Δ*Ψ*m), reactive oxygen species (ROS) production, and plasma membrane integrity. Dicentrine exhibited potent activity against promastigote and intracellular amastigote forms, with EC_50_ values ranging from 0.86 to 1.64 µg/mL. The compound showed low cytotoxicity toward macrophages (CC_50_ = 162.5 µg/mL), resulting in high selectivity indices (up to 188.3). Mechanistic studies revealed significant disruption of Δ*Ψ*m and a dose‐dependent increase in ROS production, indicating mitochondrial dysfunction and oxidative stress as key events in parasite death. These findings highlight dicentrine as a promising lead for antileishmanial drug development.

## Graphical abstract

Dicentrine, an aporphine alkaloid, exhibits potent in vitro antileishmanial activity against *Leishmania* species with high selectivity and low macrophage cytotoxicity. Mechanistic studies reveal that the compound triggers parasite death possibly by inducing mitochondrial membrane disruption and oxidative stress. These findings position dicentrine as a promising candidate for further studies in developing alternative therapies against leishmaniasis.

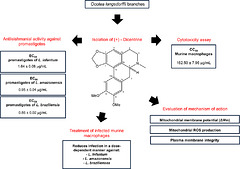

## 1 Introduction

Leishmaniasis is a neglected tropical disease complex caused by protozoan parasites of the genus *Leishmania*, transmitted to mammals through the bite of infected female sandflies. The disease manifests in three major clinical forms: cutaneous, mucosal, and visceral leishmaniasis (VL), with the latter being the most severe form, which leads to mortality rates up to 95% within 2 years if left untreated [1]. VL is highly endemic in East Africa, South Asia, and Latin America, with Brazil accounting for over 90% of cases in the Americas. In these regions, VL is primarily caused by *Leishmania (L.) infantum*, formerly known as *Leishmania chagasi*. Tegumentary leishmaniasis (TL), comprising cutaneous and mucocutaneous forms, is caused by several species, particularly *Leishmania (V.) braziliensis*, *Leishmania (L.) amazonensis*, and *Leishmania (V.) guyanensis*, depending on the geographic region. These species differ in their clinical outcomes and response to treatment, underscoring the importance of accurate species identification in endemic areas [2, 3].

Despite the global burden of leishmaniasis, current chemotherapeutic options—including pentavalent antimonials, pentamidine, amphotericin B, and miltefosine—are limited by toxicity, long treatment regimens, high cost, and the emergence of resistant strains [4]. Thus, the search for safer and more effective alternatives remains urgent. In this aspect, natural products represent an important source for antiparasitic drug discovery, with alkaloids in particular showing broad biological activities, including antimicrobial and antiparasitic effects [5]. Previous studies have highlighted the antileishmanial potential of alkaloids derived from Lauraceae plants, especially species of the genus *Ocotea*, several of which have yielded bioactive aporphine alkaloids with antiparasitic activity [6, 7, 8].

*Ocotea langsdorffii* (Meisn.) Mez (Lauraceae) is a tree species widely distributed in the Atlantic Forest and *Cerrado* (savanna‐like) biomes of Brazil. Ecologically, members of the genus *Ocotea* are important components of tropical forest ecosystems, contributing to forest structure and serving as food sources for local fauna. Ethnopharmacologically, different *Ocotea* species have been traditionally used in Brazilian folk medicine for the treatment of inflammation, pain, and infectious diseases [6, 9]. While the phytochemistry of *O. langsdorffii* itself remains largely unexplored, its close taxonomic relatives have yielded alkaloids with significant antimicrobial activity, making this species a rational candidate for bioprospecting.

Our previous work demonstrated that dicentrine, an aporphine alkaloid isolated from the branches of *Ocotea puberula*, has antileishmanial properties and affects the thermodynamic and mechanical properties of the outer plasma membrane [8, 9, 10]. While our previous report described the topical efficacy of dicentrine against cutaneous strains [10], its biological application against the visceralizing species *L. (L.) infantum* and its deep impact on the parasite's bioenergetics remained entirely unexplored. Thus, establishing *O. langsdorffii* as a novel natural source of this alkaloid expands both the chemical biodiversity and the therapeutic relevance of aporphine scaffolds.

As a continuation of our studies, the present work provides the first report of dicentrine in *O. langsdorffii* and a detailed evaluation of the in vitro activity of this alkaloid against promastigotes and intracellular amastigotes of *L. (L.) infantum*, *L. (L.) amazonensis*, and *L. (V.) braziliensis*. Furthermore, cytotoxicity was assessed in mammalian cells, and mechanistic insights were investigated by analyzing mitochondrial membrane potential, reactive oxygen species (ROS) production, and plasma membrane integrity in treated parasites. Together, these findings aim to deepen the pharmacological understanding of dicentrine and support its potential as a candidate for leishmaniasis chemotherapy.

## 2 Results

### 2.1 Chemical Characterization of Dicentrine

**FIGURE 1:**
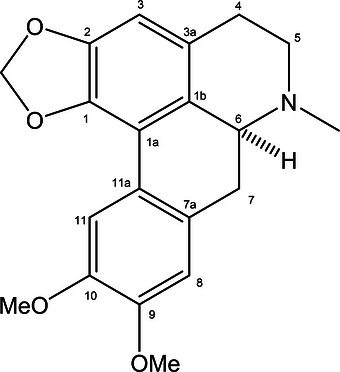
Chemical structure of dicentrine isolated from *O. langsdorffii*.

Dicentrine was isolated with 99% purity by HPLC. Its ESI‐HRMS spectrum showed [M + H]^+^ and [M + Na]^+^ ion peaks at *m/z* 340.1552 and 362.1371, respectively, consistent with the molecular formula C_20_H_21_NO_4_. Analysis of respective NMR data (Figures S1–S3 and Table S1) and optical rotation, in comparison with literature values [11], confirmed the compound as indicated in Figure 1.

### 2.2 Dicentrine Exhibits In Vitro Activity Against Leishmania ssp

Dicentrine demonstrated significant antileishmanial activity against *L. (L.) infantum*, *L. (L.) amazonensis*, and *L. (V.) braziliensis* promastigotes, with EC_50_ values of 1.64, 0.95, and 0.86 µg/mL, respectively (Table 1). For comparison, amphotericin B (AmpB) displayed EC_50_ values of 0.13, 0.25, and 0.20 µg/mL, respectively. Although AmpB was more potent overall, dicentrine showed statistically significant inhibitory activity (*p* < 0.05) across all tested species, and its EC_50_ values were particularly competitive against *L. (L.) amazonensis* and *L. (V.) braziliensis*, where it even outperformed AmpB.

**TABLE 1:** EC_50_, CC_50_ and selectivity index (SI) values of dicentrine and AmpB against *L. (L.) infantum*, *L. (L.) amazonensis*, and *L. (V.) braziliensis* promastigotes.

Compound	CC_50_ (µg/mL)	*L. (L.) infantum*		*L. (L.) amazonensis ∖*		*L. (V.) braziliensis*	
		EC_50_ (µg/mL)	SI	EC_50_ (µg/mL)	SI	EC_50_ (µg/mL)	SI
Dicentrine	162.50 ± 7.96	1.64 ± 0.08	99.1	0.95 ± 0.04	171.7	0.86 ± 0.02	188.3
AmpB	0.79 ± 0.12	0.13 ± 0.10	6.1	0.25 ± 0.10	3.2	0.20 ± 0.05	4.0

Results are shown as mean ± standard deviation of the groups. EC_50_: the concentration at which the drug exerts 50% of its maximal effect; CC_50_: the concentration of the drug that exerts 50% of cytotoxic effect; SI: selectivity index (CC_50_/EC_50_); amphotericin B: Standard drug.

Cytotoxicity assays using murine macrophages indicated a CC_50_ of 162.5 µg/mL for dicentrine, demonstrating low mammalian toxicity. By contrast, AmpB showed a CC_50_ of 0.79 µg/mL, confirming its well‐known cytotoxic profile. Selectivity index (SI) values further highlighted the favorable therapeutic window of dicentrine, with SI values of 99.1 (*L. infantum*), 171.7 (*L. amazonensis*), and 188.3 (*L. braziliensis*), compared to AmpB SI values of 6.1, 3.2, and 4.0, respectively. These results underscore dicentrine's remarkable selectivity, which could translate into a safer therapeutic profile. However, it is important to note that while dicentrine exhibited lower EC_50_ values than AmpB in some species, its clinical applicability will ultimately depend on in vivo efficacy and pharmacokinetics, which remain to be determined.

### 2.3 Dicentrine Reduces Infection in Leishmania‐Infected Macrophages

The efficacy of dicentrine against intracellular amastigotes was evaluated in infected murine macrophages. Dicentrine significantly (*p* < 0.05) reduced both the percentage of infected macrophages and the number of intracellular amastigotes in a dose‐dependent manner across all tested species. For *L. (L.) amazonensis* (Table 2), dicentrine reduced the infection rate by 11.27%, 23.98%, and 26.29% at 1, 2, and 4 µg/mL, respectively, with corresponding reductions in amastigote load of 74.24%, 81.10%, and 83.92%. In comparison, AmpB (1 µg/mL) achieved a 77.05% reduction in infection and a 73.48% reduction in amastigote burden.

**TABLE 2:** Effect of dicentrine on macrophages infected with *L. (L.) amazonensis*.

Compound	Concentration (µg/mL)	Reduction of infected macrophages (%)	Number of amastigotes per infected macrophage	Reduction of amastigotes (%)
Dicentrine	1.00	11.27	2.70 ± 0.01	74.24
	2.00	23.98	3.21 ± 0.05	81.10
	4.00	26.29	4.33 ± 0.45	83.92
AmpB	1.00	77.05	4.50 ± 0.47	73.48

Results are shown as mean ± standard deviation (SD) of triplicate determinations from two independent experiments. Different superscript letters indicate statistically significant differences compared with untreated controls (*p* < 0.05, ANOVA with Bonferroni post‐test). Amphotericin B: Standard drug.

Against *L. (V.) braziliensis* (Table 3), dicentrine showed a stronger dose‐dependent effect, reducing infection rates by 22.33%, 43.48%, and 62.23%, with amastigote reductions of 56.59%, 58.74%, and 64.71%, respectively. AmpB (1 µg/mL) produced reductions of 60.93% and 52.44% in infection rate and amastigote number, respectively, which were comparable to those obtained with dicentrine at 4 µg/mL.

**TABLE 3:** Effect of dicentrine on macrophages infected with *L. (V.) braziliensis*.

Compound	Concentration (µg/mL)	Reduction of infected macrophages (%)	Number of amastigotes per infected macrophage	Reduction of amastigotes (%)
Dicentrine	1.00	22.33	5.0 ± 0.2	56.59
	2.00	43.48	4.7 ± 0.2	58.74
	4.00	62.23	4.0 ± 0.1	64.71
AmpB	1.00	60.93	5.5 ± 0.1	52.44

Results are shown as mean ± standard deviation (SD) of triplicate determinations from two independent experiments. Superscript letters indicate statistically significant differences compared with untreated controls (*p* < 0.05, one‐way ANOVA with Bonferroni post‐test). Amphotericin B: Standard drug.

For *L. (L.) infantum* (Table 4), dicentrine reduced infection rates by 20.38%, 28.39%, and 60.85%, with corresponding amastigote reductions of 56.12%, 64.42%, and 65.95%. AmpB at 1 µg/mL reduced the infection rate by 63.01% and the amastigote number by 50.57%, demonstrating that dicentrine at 4 µg/mL matched or slightly outperformed the reference drug.

**TABLE 4:** Effect of dicentrine on macrophages infected with *L. (L.) infantum*.

Compound	Concentration (µg/mL)	Reduction of infected macrophages (%)	Number of amastigotes per infected macrophage	Reduction of amastigotes (%)
Dicentrine	1.00	20.38	5.0 ± 0.1	56.12
	2.00	28.39	4.1 ± 0.1	64.42
	4.00	60.85	3.9 ± 0.2	65.95
AmpB	1.00	63.01	5.7 ± 0.6	50.57

Results are shown as mean ± standard deviation (SD) of triplicate determinations from two independent experiments. Different superscript letters indicate statistically significant differences compared with untreated controls (*p* < 0.05, ANOVA with Bonferroni post‐test). Amphotericin B: Standard drug.

Overall, dicentrine exhibited significant dose‐dependent efficacy in reducing intracellular amastigote survival in all tested species (*p* < 0.05), with comparable or superior effects to AmpB at higher concentrations. These findings support its potential as an effective candidate for antileishmanial chemotherapy, though in vivo validation will be essential to confirm translational applicability.

### 2.4 Dicentrine Induces Mitochondrial Dysfunction and Oxidative Stress in *L. (L.) infantum* Parasites

The mechanism of action of dicentrine was explored by investigating its effects on Δ*Ψ*m, ROS generation, and plasma membrane integrity in *L. (L.) infantum* promastigotes. *Leishmania (L.) infantum* was specifically selected as the model for mechanistic studies due to its clinical significance as the primary cause of VL in the Americas, a form associated with high mortality rates when left untreated [1, 2].

**FIGURE 2:**
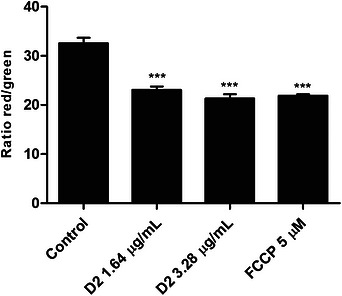
Effect of dicentrine on mitochondrial membrane potential (Δ*Ψ*m) of *L. (L.) infantum* promastigotes. Parasites (1 × 10^6^ cells/well) were kept untreated (negative control) or treated with dicentrine (1.64 and 3.28 µg/mL) and incubated for 24 h at room temperature. Cells were probed with JC‐1 fluorescent dye, and results were expressed as the ratio of red/green fluorescence intensity. Data are presented as mean ± SD of triplicate determinations (*n* = 3) from two independent experiments. Asterisks (***) indicate statistically significant differences compared with the untreated control (*p* < 0.001, one‐way ANOVA followed by Bonferroni post‐hoc test). FCCP (5 µM) was used as a positive control.

Dicentrine displayed a significant and dose‐dependent depolarization of Δ*Ψ*m in treated promastigotes at concentrations of 1.64 and 3.28 µg/mL, with reductions in the red/green fluorescence of 23.10 and 31.31, respectively, as compared to the ratio of 32.56 in the untreated control group, suggesting that dicentrine disrupts mitochondrial function. The positive control, FCCP, reduced the Δ*Ψ*m to 21.90 (Figure 2).

**FIGURE 3:**
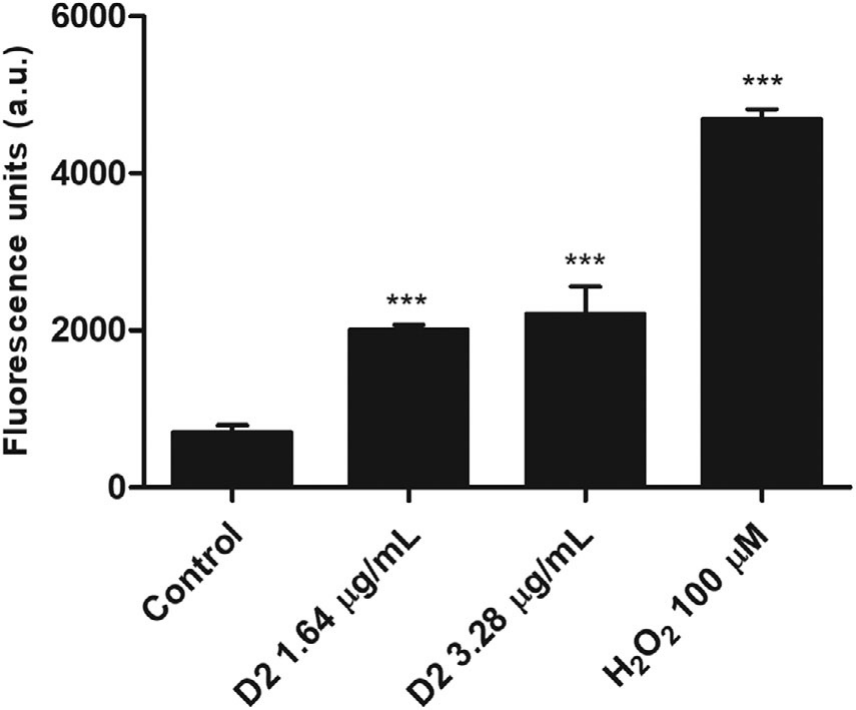
Evaluation of ROS in dicentrine‐treated *L. (L.) infantum*. Parasites (1 × 10^6^ cells/well) were kept untreated (negative control) or treated with dicentrine (1.64 and 3.28 µg/mL). After 24 h at room temperature, cells were probed with H_2_DCFDA (20 µM). The fluorescence intensity was measured by fluorimetry, and results are expressed in arbitrary units (a.u.). Data represent the mean ± SD of triplicate determinations (*n* = 3) from two independent experiments. Asterisks (***) indicate a statistically significant difference compared with the untreated control (*p* < 0.001, one‐way ANOVA with Bonferroni post‐hoc test). H_2_O_2_ (100 µM) was utilized as a positive control.

A significant increase in intracellular ROS levels was detected following dicentrine treatment. After 24 h, ROS levels increased by 183.88% and 212.46%, at concentrations of 1.64 and 3.28 µg/mL, respectively, as compared to the untreated parasites. The oxidative stress appears to be linked to the observed mitochondrial dysfunction. As expected, the positive control group treated with H_2_O_2_ exhibited an increase in the ROS levels in the order of 562.62%, as indicated in Figure 3.

**FIGURE 4:**
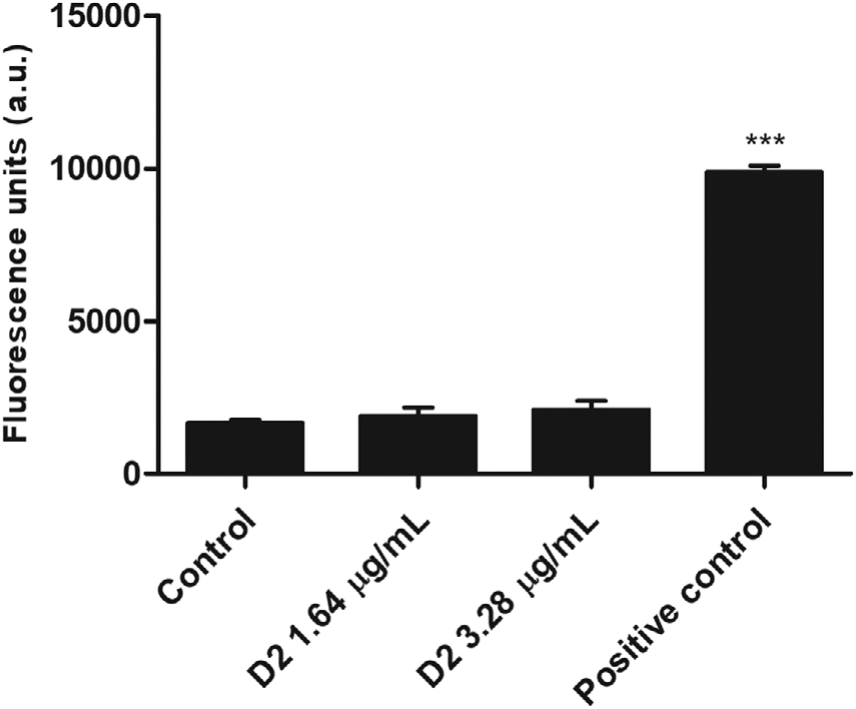
Cell membrane integrity of *L. (L.) infantum* promastigotes. Parasites (1 × 10^6^ cells/well) were kept untreated (negative control) or treated with dicentrine (1.64 and 3.28 µg/mL) for 24 h at room temperature. Cells were stained with PI (1.0 µg/mL) and evaluated by fluorimetry. Positive control: promastigotes heat‐killed at 65°C for 10 min. Data represent the mean ± SD of triplicate determinations (*n* = 3) from two independent experiments. The baseline fluorescence levels show no statistically significant difference between treated groups and the untreated control (*p* > 0.05), while asterisks (***) indicate a significant difference for the positive control (*p* < 0.0001, one‐way ANOVA with Bonferroni post‐hoc test).

To evaluate the potential for direct membrane damage induced by dicentrine, propidium iodide (PI) uptake assays were performed. Unlike the effects observed on mitochondrial function, dicentrine did not compromise plasma membrane integrity at either tested concentration. Fluorescence levels were comparable to those of the untreated controls, indicating that membrane permeability remained unaffected. This suggests that cell death is unlikely to be due to a necrotic process (Figure 4). Together, the data suggest that dicentrine exerts the antileishmanial effect through mitochondrial dysfunction and by the induction of oxidative stress, without directly disrupting plasma membrane integrity.

## 3 Discussion

Leishmaniasis is a public health concern mainly in endemic regions, since treatment options are limited and often associated with toxicity, high cost, and/or emerging drug resistance. Previous studies have reported the antiprotozoal potential of dicentrine against the amastigote forms of *L. (L.) infantum*, demonstrating an effect comparable to that of the positive control, miltefosine. Additionally, these studies revealed a plausible mechanism of action based on observed interface interactions between drugs and lipids in cell membranes, which were mimicked using Langmuir monolayers [8]. Furthermore, dicentrine showed selectivity in eliminating intracellular *L. (L.) amazonensis* and *L. (V.) braziliensis* parasites, primarily through the induction of H_2_O_2_. To evaluate its topical efficacy in vivo, a cream containing 0.5% dicentrine was tested and found to reduce both the progression of lesion size and skin parasitism [10].

Building on our previous studies with dicentrine, the present work focuses on the first isolation of this aporphine alkaloid from the Brazilian plant *O. langsdorffii*, the evaluation of its antileishmanial activity against parasite species responsible for both cutaneous and visceral leishmaniasis (TL and VL), and the investigation of its mechanism of action in *L. (L.) infantum* parasites. Our findings demonstrated that dicentrine exhibits potent activity against the *L. (L.) infantum*, *L. (L.) amazonensis*, and *L. (V.) braziliensis* promastigotes, with all EC_50_ values below 2 µg/mL. Notably, the molecule exhibited a remarkably high selectivity index, ranging from 99.1 to 188.3, indicating then a strong selectivity for parasites over host cells. Compared to AmpB, dicentrine showed higher SI values, positioning it as a promising antileishmanial candidate for further development. These results align with previous studies showing the potent antiprotozoal activity of aporphine alkaloids, such as dicentrine, via mechanisms including mitochondrial targeting and oxidative stress induction [12, 13]. The intracellular efficacy of dicentrine was evaluated using infected macrophages. Initially, treatment with dicentrine significantly reduced the *L. (L.) amazonensis‐* and *L. (V.) braziliensis‐*infected cell percentage and the number of intracellular amastigotes. Results suggest that dicentrine can limit parasite replication in parasite promastigotes and effectively target the clinically relevant amastigote stage inside host cells—an essential feature for any candidate compound aimed at treating leishmaniasis.

Mechanistically, dicentrine was shown to compromise mitochondrial function in the parasites, as evidenced by a significant decrease in Δ*Ψ*m. The depolarization was dose‐dependent and coincided with an increase in ROS production, suggesting that dicentrine induces mitochondrial stress, which leads to parasite death. Interestingly, plasma membrane integrity remained intact, implying that dicentrine may trigger a pathway of apoptotic‐like death rather than necrosis, as has been proposed for other natural products that target *Leishmania* mitochondria [14, 15, 16].

The physiological stress observed after dicentrine treatment mirrors the effects of established leishmanicidal drugs that target cellular bioenergetics. Unlike mammalian cells, *Leishmania* parasites possess a unique, single mitochondrion, rendering them exceptionally vulnerable to electron transport chain disruptions. The increase in intracellular ROS, combined with the collapse of Δ*Ψ*m, points to a cascade of apoptotic‐like events, which prevents the necrotic leakage of intracellular contents and host tissue injury.

Mitochondria play a crucial role in the bioenergetics and redox homeostasis of *Leishmania*, making them a validated target for antiparasitic agents. Mitochondrial depolarization is an early event in programmed cell death in protozoa and is often accompanied by ROS generation and downstream signaling, which leads to DNA fragmentation and metabolic arrest [17, 18]. The substantial increase in ROS levels observed after dicentrine treatment aligns with this model and could exacerbate oxidative damage, contributing to parasite death. Importantly, the preservation of host cell membrane integrity after treatment highlights dicentrine's favorable safety profile. This characteristic is critical in therapeutic development because many current treatments, such as pentavalent antimonials and AmpB, have significant adverse effects and cytotoxicity [19]. Although AmpB is widely used in the treatment of VL, its clinical use is limited by its significant toxicity, especially nephrotoxicity and hepatotoxicity, in addition to the high cost of liposomal formulations [20, 21, 22]. In contrast, dicentrine demonstrated anti‐leishmanial activity comparable to AmpB in vitro, with lower toxicity in host cells and a promising action profile on parasite mitochondria, including loss of membrane potential and increased generation of ROS, indicating its potential as a safer and more economically viable alternative in the development of new leishmanicidal drugs. It is important to highlight that more studies are necessary to test the efficacy of dicentrine in in vivo studies with different concentrations and treatment regimens to evaluate the potential of using this molecule as a possible treatment against leishmaniasis.

## 4 Conclusion

In this study, the aporphine alkaloid dicentrine was isolated for the first time from the branches of *O. langsdorffii* (Lauraceae), and its effects against *Leishmania (L.) infantum, L. (L.) amazonensis*, and *L. (V.) braziliensis* were evaluated. Although some of its antileishmanial effects have been previously reported, we demonstrate for the first time that dicentrine induces mitochondrial disruption and promotes the accumulation of ROS in parasite promastigotes. These findings corroborate our previous studies describing the potential interactions between dicentrine and biomimetic cell membrane models. Based on these in vitro findings, dicentrine displays selective antileishmanial activity against the tested species and low toxicity toward murine macrophages. However, these data are preliminary, and future in vivo studies, including pharmacokinetic evaluations and comprehensive preclinical toxicity validations, are strictly necessary to confirm the therapeutic potential and safety profile of this aporphine alkaloid.

## 5 Experimental

### 5.1 General Experimental Procedure

Silica gel (60–210 µm, Merck) was used for column chromatography, and silica gel F_254_ (Merck) was used for analytical thin‐layer chromatography (TLC). High‐performance liquid chromatography (HPLC) analyses were performed using a Thermo Scientific Ultimate 3000 Diode Array Detector (DAD) with a Phenomenex Luna C_18_ column (5 µm, 250 × 4.6 mm; flow rate: 1.0 mL/min). Optical rotation was measured on a JASCO DIP‐370 digital polarimeter (Na filter, *λ* = 588 nm). NMR spectra were recorded on a Varian INOVA spectrometer operating at 500 MHz (^1^H) and 125 MHz (^1^ ^3^C), using CDCl_3_ as the solvent and TMS as the internal standard. ESI‐HRMS spectrum was measured using a Bruker Daltonics QTOF MAXIS 3G spectrometer with electrospray ionization in positive mode.

### 5.2 Plant Material

Branches of *O. langsdorffii* were collected in April 2022 in Santana do Riacho, Minas Gerais State, Brazil. The plant material was identified by Dr. Guilherme M. Antar and registered in the SISGEN database under code A4123E4. A voucher specimen was deposited in the Herbarium of the University of São Paulo (SPF) under the number SPF 4222.

### 5.3 Extraction and Isolation

Dried and ground branches of *O. langsdorffii* (250 g) were defatted with hexane (6 × 250 mL), basified with NH_3_.H_2_O (pH 10.0), and subsequently extracted with CH_2_Cl_2_ (4 × 250 mL). The resulting CH_2_Cl_2_ extract was acidified with HCl (pH 2.0), and the aqueous layer was then basified with NH_3_.H_2_O. This was followed by reextraction with CH_2_Cl_2_ (4 × 50 mL). The combined organic phases were dried over anhydrous MgSO_4_, and the solvent was removed under reduced pressure to yield 113 mg of an alkaloid fraction. As this fraction exhibited potent activity against *L. (L.) amazonensis, L. (V.) braziliensis*, and *L. (L.) infantum* (100% parasite mortality at 200 µg/mL), a portion of the material (100 mg) was subjected to silica gel column chromatography and eluted with increasing amounts of EtOAc in CH_2_Cl_2_, yielding 14 fractions (A–N). Following evaluation of their antiparasitic activities, only fraction J (55.1 mg) retained activity against all three tested *Leishmania* species, causing 100% parasite mortality at 200 µg/mL. This fraction was subsequently purified by silica gel column chromatography using a gradient of EtOAc in CH_2_Cl_2_, affording 8.6 mg of pure dicentrine.

(+*)‐Dicentrine*. [α]_D_^24^ + 72.7 (*c* 0.0005, MeOH). ^1^H NMR (CDCl_3_, 500 MHz): *δ* 7.67 (s, H‐11), 6.78 (s, H‐8), 6.52 (s, H‐3), 6.07 (s, OCH_2_O), 5.92 (s, OCH_2_O), 3.92 (s, 9‐ and 10‐OCH_3_), 3.16 – 3.03 (m, H‐4, H‐5, H‐6a, H‐7), 2.55 (s, NCH_3_), 2.65 ‐ 2.60 (m, H‐4, H‐5, H‐7). ^13^C NMR (CDCl_3_, 125 MHz): *δ* 148.2 (C‐10), 147.6 (C‐9), 146.6 (C‐1), 141.7 (C‐2), 128.3 (C‐7a), 126.5 (C‐3a), 126.3 (C‐1b), 123.5 (C‐11a), 116.5 (C‐1a), 111.3 (C‐8), 110.6 (C‐11), 106.7 (C‐3), 100.6 (OCH_2_O), 62.3 (C‐6a), 56.1 (10‐OCH_3_), 55.8 (9‐OCH_3_), 53.5 (C‐5), 43.8 (NCH_3_), 34.1 (C‐7), 29.1 (C‐4). ESI‐HRMS *m/z* 340.1552 [M + H]^+^ (calcd. for C_20_H_22_NO_4,_ 340.1549) and *m/z* 362.1371 [M + Na]^+^ (calcd. for C_20_H_21_NO_4_Na, 362.1368).

### 5.4 Ethical Compliance and Parasites

The experiments adhered to ethical guidelines, and the study was approved by the Ethical Committee for Animal Research at the Federal University of Minas Gerais (UFMG), under protocol number 056/2022. Female BALB/c mice, aged 6 weeks, were obtained from the UFMG Bioterium Center (Central Animal Facility of UFMG, Belo Horizonte, Brazil; RRID: IMSR_JAX:000651), and maintained under specific pathogen‐free conditions. The primary mammalian cells were freshly isolated from female BALB/c mice following well‐established protocols approved by the local Ethics Committee. To elicit the macrophages, mice received an intraperitoneal injection of 3.0 mL of a 3% (w/v) sterile thioglycolate broth (Sigma–Aldrich, St. Louis, MO, USA). At 72 h post‐injection, the animals were euthanized, and the peritoneal exudate cells were harvested by washing the peritoneal cavity with 5.0 mL of ice‐cold RPMI 1640 medium.

*Leishmania (L.) amazonensis* (IFLA/BR/1967/PH‐8), *L. (V.) braziliensis* (MHOM/BR/1975/M2903), and *L. (L.) infantum* (MHOM/BR/1970/BH46) strains were cultivated at 24°C in Schneider's medium. The medium was enriched with 20% heat‐inactivated fetal bovine serum (FBS) (v/v), 20 mM L‐glutamine, penicillin (200 U/mL), streptomycin (100 µg/mL), and gentamicin (50 µg/mL) to maintain a pH of 7.4 [23].

### 5.5 Antileishmanial Activity and Cytotoxicity Assessment of Dicentrine

To determine the 50% effective concentration (EC_50_) against stationary‐phase promastigotes of *L. (L.) infantum, L. (V.) amazonensis*, and *L. (L.) braziliensis*, parasites (1 × 10^6^ cells/well) were incubated with dicentrine (0–200 µg/mL) or amphotericin B (AmpB; 0–10 µg/mL) in 96‐well plates. For bioactivity‐guided fractionation, the samples were evaluated at a single concentration of 200 µg/mL against the same parasite species. The cultures were kept for 48 h at 24°C, when the cell viability was assessed through an MTT assay [24]. The optical density (OD) values were measured at 570 nm in a SpectraMax Plus microplate spectrophotometer (Molecular Devices). EC_50_ values were calculated using sigmoidal regression analysis with Microsoft Excel (version 10.0) and GraphPad Prism (version 10.0.2).

The cytotoxic concentration at 50% (CC_50_) of dicentrine was evaluated in primary murine peritoneal macrophages (5 × 10^6^ cells/mL), which were cultured in RPMI 1640 medium supplemented with FBS and L‐glutamine. Cells were incubated with dicentrine (0–200 µg/mL) or AmpB (0–10 µg/mL) for 48 h at 37°C. Macrophage viability was determined through an MTT assay, and CC_50_ values were calculated from dose–response curves via sigmoidal regression using the aforementioned software. SIs were calculated as the ratio of CC_50_ to EC_50_ [25].

### 5.6 Treatment of Infected Macrophages

The efficacy of dicentrine against intracellular *Leishmania* amastigotes was evaluated using an in vitro macrophage infection model. First, primary murine macrophages (5 × 10^6^ cells/mL) were seeded onto glass coverslips in an RPMI 1640 medium, which was enriched with 20% FBS, L‐glutamine 20 mmol/L, penicillin 200 U/mL, streptomycin 100 µg/mL e gentamicin 50 µg/mL. The cultures were incubated for 2 h at 37°C in an atmosphere with 5% CO_2_. Then, stationary promastigotes were added to the cultures at a ratio of 10:1 parasite:cell, and incubated for 24 h at 37°C. After that, unattached parasites were removed by washing, and infected macrophages were treated with dicentrine (1, 2 and 4 µg/mL) or AmpB (at a concentration of 1 µg/mL) for 48 h at 37°C. Untreated and uninfected macrophages were used as controls. After treatment, cells were fixed with 4% paraformaldehyde and stained with the Panoptic method. Infection rate and intracellular amastigote burden were determined microscopically by manually counting 200 macrophages per coverslip. Counts were performed in triplicate by two independent, blind observers [25].

### 5.7 Evaluation of Membrane Mitochondrial Potential (Δ*Ψ*m) in Parasites

The following experiments were conducted based on the value of EC_50_ in *L. (L.) infantum* to determine the concentrations used: one time the EC_50_ value and two times the EC_50_ value. After treatment with dicentrine at concentrations of 1.64 and 3.28 µg/mL, *L. infantum* promastigotes were labeled with 5 µg/mL of JC‐1 (Sigma–Aldrich, St. Louis, MO, USA) in the dark for 30 min, at 37°C. Next, parasites were washed with Hanks' balanced salt solution (HBSS), and plates were read in a spectrofluorimeter microplate reader (FLx800, BioTek Instruments, Inc., Winooski, VT, USA) using excitation and emission wavelengths of 485/428 nm and 540/600 nm, respectively [25].

### 5.8 Measurement of Mitochondrial ROS Production in Parasites

After treatment with dicentrine at concentrations of 1.64 and 3.28 µg/mL, *L. (L.) infantum* promastigotes adjusted to 5 × 10^6^ cells per well, and H_2_DCFDA (2', 7'‐dichlorodihydrofluorescein diacetate, Molecular Probes, Eugene, OR, USA) was added, at a concentration of 20 µM. Samples were incubated in the dark for 30 min at room temperature. The fluorescence intensity was evaluated using a spectrofluorimeter (FLx800, BioTek Instruments, Inc., Winooski, VT, USA), with excitation and emission wavelengths of 485 and 528 nm, respectively [26].

### 5.9 Evaluation of Plasma Membrane Integrity in Parasites

After treatment with dicentrine at concentrations of 1.64 and 3.28 µg/m, *L. (L.) infantum* promastigotes were adjusted to 5 × 10^6^ cells per well. Then, PI (1.0 µg/mL; Sigma–Aldrich, USA) was added, and samples were incubated for 15 min at room temperature. They were read in a spectrofluorimeter (FLx800, BioTek Instruments, Inc., Winooski, VT, USA), at excitation and emission wavelengths of 540 and 600 nm, respectively [26].

### 5.10 Statistical Analysis

Statistical analyses were performed using GraphPad Prism software (version 10.0.2, GraphPad Software Inc., San Diego, CA, USA). Data pre‐processing involved testing for normal distribution using the Shapiro–Wilk normality test, and homoscedasticity was verified. Quantitative data are presented as the mean ± standard deviation (SD) of triplicate determinations (*n* = 3) collected from two independent experimental assays. Statistical significance was assessed by ANOVA followed by the Bonferroni post‐hoc test for multiple comparisons (two‐sided testing; testing level alpha = 0.05). *p* values less than 0.05 (*p* < 0.05) were considered statistically significant.

## Author Contribution

**Camila S. de Freitas**: writing—original draft, methodology, investigation, formal analysis. **Eduardo Oliveira S. Da Silva**: investigation, formal analysis. **Daniela C. Tristão**: methodology, investigation. **Guilherme M. Antar**: methodology, investigation. **Daniela P. Lage**: methodology, investigation. **Dóris M. Abrão**: methodology, investigation. **Luciana M. Ribeiro Antinarelli**: methodology, investigation. **Elaine S. Coimbra**: methodology, investigation. **João Henrique G. Lago**: writing—original draft, supervision, funding acquisition, formal analysis, data curation, conceptualization. **Eduardo A.F. Coelho**: writing—original draft, supervision, funding acquisition, formal analysis, data curation, conceptualization.

## Funding

This work was supported by Fundação de Amparo a Pesquisa do Estado de São Paulo, Fundação de Amparo a Pesquisa do Estado de Minas, Coordenação de Aperfeiçoamento de Pessoal de Nível Superior.

## Ethics Statement

All procedures involving animals were conducted in accordance with institutional guidelines and approved by the Institutional Animal Care and Use Committee (CEUA protocol no. 056/2022).

## Conflicts of Interest

The authors declare no conflicts of interest.

## Supporting information




**Supporting File 1**: cbdv71575‐sup‐0001‐SupMat.docx

## Data Availability

The data that support the findings of this study are available from the corresponding author upon reasonable request.

## References

[1] S. Burza , S. L. Croft , and M. Boelaert , “Leishmaniasis,” Lancet 392 (2018): 951–970, 10.1016/S0140-6736(18)31204-2. 30126638

[2] World Health Organization , ‘Leishmaniasis: Fact Sheets’, (2023): https://www.who.int/news‐room/fact‐sheets/detail/leishmaniasis.

[3] J. Alvar , I. D. Vélez , C. Bern , et al., “Leishmaniasis Worldwide and Global Estimates of Its Incidence,” PLoS ONE 7 (2012): e35671, 10.1371/journal.pone.0035671. 22693548 PMC3365071

[4] A. Ponte‐Sucre , F. Gamarro , J. C. Dujardin , et al., “Drug Resistance and Treatment Failure in Leishmaniasis: A 21st Century Challenge,” PLOS Neglected Tropical Diseases 11 (2017): e0006052, 10.1371/journal.pntd.0006052. 29240765 PMC5730103

[5] X. Shang , L. Dai , X. Cao , et al., “Natural Products in Antiparasitic Drug Discovery: Advances, Opportunities and Challenges,” Natural Product Reports 42 (2025): 1419–1458, 10.1039/D5NP00007F. 40501439

[6] H. Barbosa , R. L. C. G. da Silva , T. A. Costa‐Silva , et al., “Interaction of Dicentrinone, an Antitrypanosomal Aporphine Alkaloid Isolated From Ocotea puberula (Lauraceae), in Cell Membrane Models at the Air‐Water Interface,” Bioorganic Chemistry 101 (2020): 103978, 10.1016/j.bioorg.2020.103978. 32534347

[7] H. Barbosa , T. A. Costa‐Silva , G. A. Alves Conserva , et al., “Aporphine Alkaloids From Ocotea puberula With Anti‐ Trypanosoma Cruzi Potential—Activity of Dicentrine‐β‐ N ‐Oxide in the Plasma Membrane Electric Potentials,” Chemistry & Biodiversity 18 (2021): e2001022, 10.1002/cbdv.202001022. 33635585

[8] M. E. Rosa , D. C. Tristão , H. Barbosa , et al., “Exploring the Antileishmanial Activity of Dicentrine From Ocotea puberula (Lauraceae) Using Biomembrane Models,” Bioorganic Chemistry 147 (2024): 107408, 10.1016/j.bioorg.2024.107408. 38678776

[9] M. P. Correia , Dicionário de Plantas úteis do Brasil e das Exóticas Cultivadas (Imprensa Nacional, 1984).

[10] J. A. Jesus , G. V. Araujo Flores , D. C. D. S. Souza , et al., “Dicentrine Purified From the Leaves of Ocotea Puberula Controls the Intracellular Spread of L. (L.) amazonensis and L. (V.) braziliensis Amastigotes and Has Therapeutic Activity as a Topical Treatment in Experimental Cutaneous Leishmaniasis,” Microorganisms 13 (2025): 309, 10.3390/microorganisms13020309. 40005676 PMC11858304

[11] W. G. Ma , Y. Fukushi , S. Tahara , and T. Osawa , “Fungitoxic Alkaloids From Hokkaido Papaveraceae,” Fitoterapia 71 (2000): 527–534, 10.1016/S0367-326X(00)00193-3. 11449501

[12] D. C. Soares , N. A. Portella , M. F. S. Ramos , A. C. Siani , and E. M. Saraiva , “Trans‐β‐caryophyllene: An Effective Antileishmanial Compound Found in Commercial Copaiba Oil (Copaifera spp.),” Evidence Based‐Complementary and Alternative Medicine 2013 (2013): 761323. 23864897 10.1155/2013/761323 PMC3705974

[13] R. Essid , F. Z. Rahali , K. Msaada , et al., “Antileishmanial and Cytotoxic Potential of Essential Oils From Medicinal Plants in Northern Tunisia,” Industrial Crops and Products 77 (2015): 795–802, 10.1016/j.indcrop.2015.09.049.

[14] A. G. Tempone , P. Sartorelli , D. Teixeira , et al., “Brazilian Flora Extracts as Source of Novel Antileishmanial and Antifungal Compounds,” MemÃ3rias do Instituto Oswaldo Cruz 103 (2008): 443–449, 10.1590/S0074-02762008000500006. 18797756

[15] M. C. Duarte , G. S. V. Tavares , D. G. Valadares , et al., “Antileishmanial Activity and Mechanism of Action From a Purified Fraction of Zingiber officinalis Roscoe Against Leishmania amazonensis,” Experimental Parasitology 166 (2016): 21–28, 10.1016/j.exppara.2016.03.026. 27013260

[16] W. S. Koko , I. S. Al Nasr , T. A. Khan , R. Schobert , and B. Biersack , “An Update on Natural Antileishmanial Treatment Options From Plants, Fungi and Algae,” Chemistry & Biodiversity 19 (2022): e202100542, 10.1002/cbdv.202100542. 34822224

[17] F. J. Pérez‐Victoria , S. Castanys , and F. Gamarro , “Leishmania donovani Resistance to Miltefosine Involves a Defective Inward Translocation of the Drug,” Antimicrobial Agents and Chemotherapy 47 (2003): 2397–2403, 10.1128/AAC.47.8.2397-2403.2003. 12878496 PMC166066

[18] H. P. C. Brígido , L. G. A. Santos , R. C. Barros , et al., “The Role of Oxidative Stress in the Pathogenesis and Treatment of Leishmaniasis: Impact on Drug Toxicity and Therapeutic Potential of Natural Products,” Toxics 13 (2025): 190. 40137517 10.3390/toxics13030190 PMC11945835

[19] H. Zhang , R. Yan , Y. Liu , et al., “Progress in Antileishmanial Drugs: Mechanisms, Challenges, and Prospects,” PLOS Neglected Tropical Diseases 19 (2025): e0012735, 10.1371/journal.pntd.0012735. 39752369 PMC11698350

[20] S. Sundar and J. Chakravarty , “Liposomal Amphotericin B and Leishmaniasis: Dose and Response,” Journal of Global Infectious Diseases 2 (2010): 159, 10.4103/0974-777X.62886. 20606972 PMC2889656

[21] B. Monge‐Maillo and R. López‐Vélez , “Therapeutic Options for Visceral Leishmaniasis,” Drugs 74 (2013): 1683–1700. 10.1007/s40265-013-0133-0 PMC3837193 24170666

[22] J. Berman , “Amphotericin B Formulations and Other Drugs for Visceral Leishmaniasis,” American Society of Tropical Medicine and Hygiene 92 (2015): 471–473, 10.4269/ajtmh.14-0743.

[23] E. A. F. Coelho , C. A. P. Tavares , F. A. A. Carvalho , et al., “Immune Responses Induced by the Leishmania ( Leishmania ) donovani A2 Antigen, but Not by the LACK Antigen, are Protective Against Experimental Leishmania ( Leishmania ) amazonensis Infection,” Infection and Immunity 71 (2003): 3988–3994, 10.1128/IAI.71.7.3988-3994.2003. 12819086 PMC162020

[24] T. Mosmann , “Rapid Colorimetric Assay for Cellular Growth and Survival: Application to Proliferation and Cytotoxicity Assays,” Journal of Immunological Methods 65 (1983): 55–63, 10.1016/0022-1759(83)90303-4. 6606682

[25] C. S. Freitas , S. S. Santiago , D. P. Lage , et al., “In Vitro Evaluation of Antileishmanial Activity, Mode of Action and Cellular Response Induced by Vanillin Synthetic Derivatives Against Leishmania Species Able to Cause Cutaneous and Visceral Leishmaniasis,” Experimental Parasitology 251 (2023): 108555, 10.1016/j.exppara.2023.108555. 37247802

[26] F. S. D. Santos , R. P. D. Freitas , C. S. D. Freitas , et al., “Synthesis of 1,2,3‐triazole‐Containing Methoxylated Cinnamides and Their Antileishmanial Activity Against the Leishmania Braziliensis Species,” Pharmaceuticals 16 (2023): 1113, 10.3390/ph16081113. 37631028 PMC10459042

