# Supplementary material for: Dicentrine, an Aporphine Alkaloid From Ocotea langsdorffii, Induces Mitochondrial Dysfunction and Oxidative Stress in Leishmania (L.) infantum
Source: Chem Biodivers. 2026 Aug 9;23(8):e71575. doi: 10.1002/cbdv.71575 (PMC13453212; doi:10.1002/cbdv.71575)
Supplement: Supplementary file 1 [file CBDV-23-e71575-s001.docx]

Dicentrine, an Aporphine Alkaloid from *Ocotea langsdorffii*, Induces Mitochondrial Dysfunction and Oxidative Stress in *Leishmania (L.)* *infantum*

Camila S. de Freitas^a *^, Eduardo Oliveira S. da Silva^b^, Daniela C. Tristão^b^,

Guilherme M. Antar^c^, Daniela P. Lage^a^, Dóris M. Abrão^a^, Luciana M. Ribeiro Antinarelli^d^, Elaine S. Coimbra^d^, João Henrique G. Lago^b^, Eduardo A.F. Coelho^a^

^a^ Post-graduation Program in Health Sciences - Infectiology and Tropical Medicine, Medicine Faculty, Federal University of Minas Gerais, Belo Horizonte, MG, 30130-100, Brazil.

^b^ Center of Natural Sciences and Humanities, Federal University of the ABC, Santo Andre, SP, 09210-180, Brazil.

^c^ Department of Agricultural and Biological Sciences, Federal University of Espirito Santo, São Matheus, ES, 29932-540, Brazil.

^d^ Departament of Parasitology, Microbiology and Imunology, Institute of Biological Sciences, Federal University of Juiz de Fora, Juiz de Fora, MG, 36036‐900, Brazil

***** Corresponding author

E-mail address: camilasimoesf@gmail.com

**SUPPLEMENTARY INFORMATION**

**Table S1** - ^1^H and ^13^C NMR data to dicentrine

(δ/ppm, 500 e 125 MHz, CDCl_3_)

|  | **^1^H (mult., *J*/Hz)** | | **^13^C** | |
| --- | --- | --- | --- | --- |
| **Position** | **Experimental** | **Literature*** | **Experimental** | **Literature*** |
| **1** | - | - | 146.6 | 146.2 |
| **1a** | - | - | 116.5 | 116.2 |
| **1b** | - | - | 126.3 | 126.3 |
| **2** | - | - | 141.7 | 141.4 |
| **3** | 6.52 (s) | 6.51 (s) | 106.7 | 106.2 |
| **3a** | - | - | 126.5 | 126.6 |
| **4** | 3.16 – 3.03 (m)  2.65 – 2.60 (m) | 3.16 (m)  2.64 (br d, 14.0) | 29.1 | 28.9 |
| **5** | 3.16 – 3.03 (m)  2.65 – 2.60 (m) | 3.07 (m) 2.56 (m) | 53.5 | 53.2 |
| **6a** | 3.16 – 3.03 (m) | 3.23 (br d, 10.0) | 62.3 | 62.1 |
| **7** | 3.16 – 3.03 (m)  2.65 – 2.60 (m) | 3.07 (dd, 15.0, 4.5)  2.67 (dd, 15.0, 10.0) | 34.1 | 33.8 |
| **7a** | - | - | 128.3 | 128.2 |
| **8** | 6.78 (s) | 6.79 (s) | 111.3 | 111.2 |
| **9** | - | - | 147.6 | 147.5 |
| **10** | - | - | 148.2 | 148.2 |
| **11** | 7.67 (s) | 7.63 (s) | 110.6 | 110.4 |
| **11a** | - | - | 123.5 | 123.1 |
| **9-OCH_3_** | 3.92 (s) | 3.93 (s) | 55.8 | 55.4 |
| **10-OCH_3_** | 3.92 (s) | 3.93 (s) | 56.1 | 55.6 |
| **OCH_2_O** | 5.92 (s)  6.07 (s) | 5.93 (s)  6.05 (s) | 100.6 | 100.4 |
| **NCH_3_** | 2.55 (s) | 2.58 (s) | 43.8 | 43.3 |

*W. G. Ma, Y. Fukushi, S. Tahara, T. Osawa. ‘Fungitoxic alkaloids from Hokkaido Papaveraceae’ *Fitoterapia* **2000**, *71*, 527-34.

**Figure S1.** ESI-HRMS spectrum (positive mode) of dicentrine.

**Figure S2.** ^1^H NMR spectrum of dicentrine (δ, 500 MHz, CDCl_3_).

**Figure S3.** ^13^C NMR spectrum of dicentrine (δ, 125 MHz, CDCl_3_).
